# Hemispherically-Unified Surface Maps of Human Cerebral Cortex: Reliability and Hemispheric Asymmetries

**DOI:** 10.1371/journal.pone.0045582

**Published:** 2012-09-18

**Authors:** Xiaojian Kang, Timothy J. Herron, Anthony D. Cate, E. William Yund, David L. Woods

**Affiliations:** 1 Human Cognitive Neurophysiology Lab, VA Research Service, Department of Veterans Affairs Medical Center, Martinez, California, United States of America; 2 Department of Neurology and Center for Neuroscience, Sacramento, California, United States of America; 3 UC Davis Center for Mind and Brain, Davis, California, United States of America; Beijing Normal University, Beijing, China

## Abstract

Understanding the anatomical and structural organization of the cerebral cortex is facilitated by surface-based analysis enabled by FreeSurfer, Caret, and related tools. Here, we examine the precision of FreeSurfer parcellation of the cortex and introduce a method to align FreeSurfer-registered left and right hemispheres onto a common template in order to characterize hemispheric asymmetries. The results are visualized using Mollweide projections, an area-preserving map. The regional distribution, inter-hemispheric asymmetries and intersubject variability in cortical curvature, sulcal depth, cortical thickness, and cortical surface area of 138 young, right handed subjects were analyzed on the Mollweide projection map of the common spherical space. The results show that gyral and sulcal structures are aligned with high but variable accuracy in different cortical regions and show consistent hemispheric asymmetries that are maximal in posterior temporal regions.

## Introduction

Inflation of the highly convoluted cerebral cortex to a simple smooth surface such as a sphere or flat map is a powerful tool for elucidating the functional organization and anatomical structure of human cortex [1], [2]. Aligning functional data to the gyral and sulcal structures of the cortical surface permits the visualization of the organization of visual [3]–[5], somatosensory [6], motor [7], and auditory cortex [8], [9]. In addition, in functional neuroimaging studies cortical surface analysis improves the magnitude and significance of functional activations in comparison with analyses performed in 3D (volumetric) space [2], [10]–[12].

Surface-based anatomical studies of human cerebral cortex have also been used to analyze cortical anatomy including cortical folding patterns [13], gray matter volume [14], [15], cortical tissue properties [16], and thickness and regional area [17]–[19]. The use of surface-based alignment also increases the power and precision of detecting cortical abnormalities [20]–[24] and studying longitudinal anatomical changes [25], [26].

Fischl et al. [1] introduced FreeSurfer, a whole-hemisphere surface-based technique that permits the automatic across-subject averaging of data from individual subjects in three steps: (1) inflating each hemispheric surface to a sphere, (2) aligning hemisphere structures from individual subjects with the average convexity templates of the left hemisphere (LH) or right hemisphere (RH), and (3) fine tuning the sulcal alignment using local curvature. In the current manuscript we analyzed the accuracy of FreeSurfer across-subject alignment in different regions of each hemisphere. The results show that cortical surface structures are aligned with high but variable accuracy in different cortical regions.

One limitation of Freesurfer is that the spherical maps of the LH and RH surfaces are not aligned, complicating interhemispheric comparisons of anatomical and functional properties [27]–[30]. Although FreeSurfer provides a standard method (contra-surface coregistration) to coregister the LH to the RH template non-linearly, and vice-versa [31], such a procedure reduces the accuracy of the alignment of the “contra-registered” hemisphere and biases results based on which hemisphere template is selected [30] (see the Method section for further discussion).

We therefore propose a simple method to align the averaged FreeSurfer registered spheres of the LH and RH across all subjects by rigid-body spherical transformation in spherical space. This produces a common, unbiased coordinate sphere by averaging the aligned mean LH and mirrored RH. The same, fixed alignment parameters from the averaged LH and mirrored RH were applied to align the LH and mirrored RH of 138 individual subjects. This approach revealed small but significant asymmetries in surface curvature, sulcal depth, cortical thickness, and area of corresponding parcellations of the two hemispheres with the largest differences seen in peri-Sylvian and posterior temporal cortex.

## Materials and Methods

We studied 138 young, well-educated, right-handed subjects including age-matched groups of 69 females (ages 18–38 years, mean 26.3 years) and 69 males (ages 18–38 years, mean 26.1 years) matched in education (males  = 15.0, females  = 15.1 yrs). Ethics approval for the study was obtained from the Institutional Review Board of the Northern California Health Care System within the US Department of Veterans Affairs. Informed, written consent was obtained from all of the subjects.

**Figure 1:**
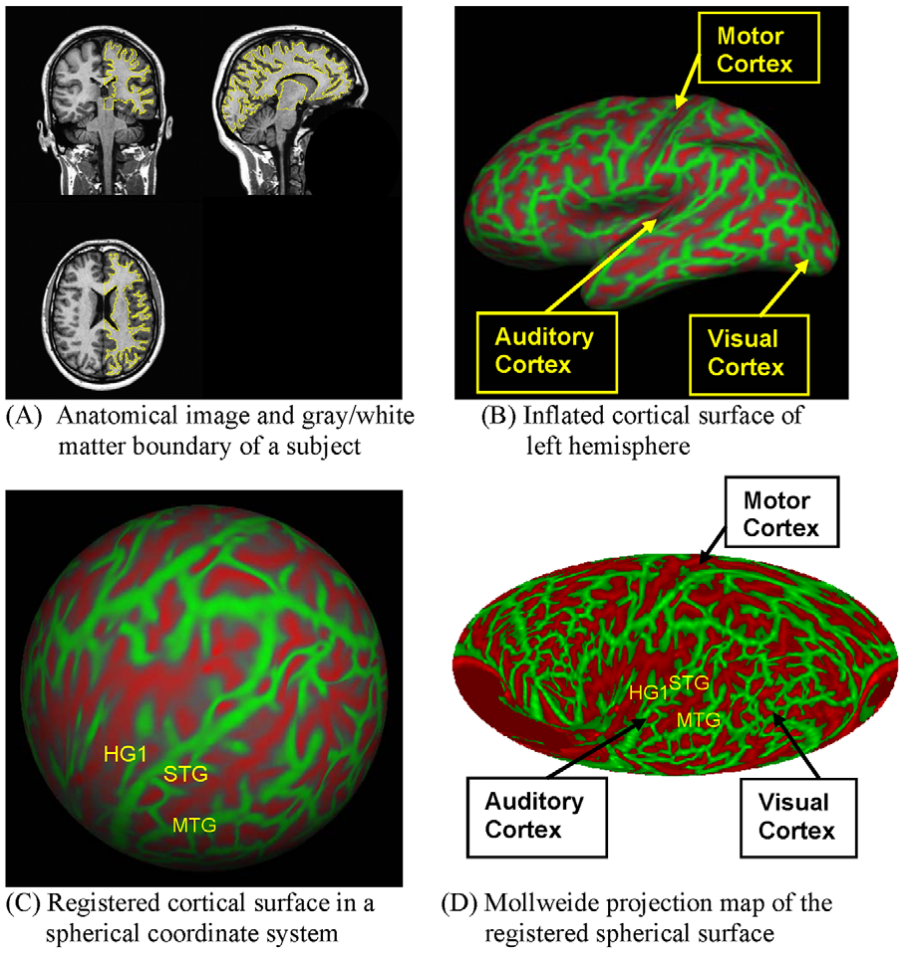
Inflation, registration and projection of the GM/WM boundary for a subject. GM/WM surface convexity is color-coded (Gyri  =  green, sulci  =  red). (A) Anatomical image and GM/WM boundary of a subject; (B) Inflated GM/WM boundary of left hemisphere; (C) Registered GM/WM surface in a spherical coordinate system; (D) Mollweide projection map of the registered spherical surface. HG1  =  anterior Heschl's gyrus; MTG  =  middle temporal gyrus; STG  =  superior temporal gyrus.

Two high-resolution T1 anatomical images (TR  = 15 ms, TE  = 4.47 ms, Flip Angle  = 35°, voxel size 0.94×1.30×0.94 mm) were acquired on a 1.5 T Philips Eclipse scanner. These anatomical images were re-sampled to 1×1×1 mm resolution, averaged, and then inflated to the cortical surface using FreeSurfer [32], [33]. The inflated cortical surfaces of LH and RH were then co-registered to a spherical coordinate system [1] based on reference templates for each hemisphere derived from the average pattern of 40 individual subjects. Figure 1A–C shows the procedure of cortical surface segmentation, inflation and co-registration.

**Figure 2:**
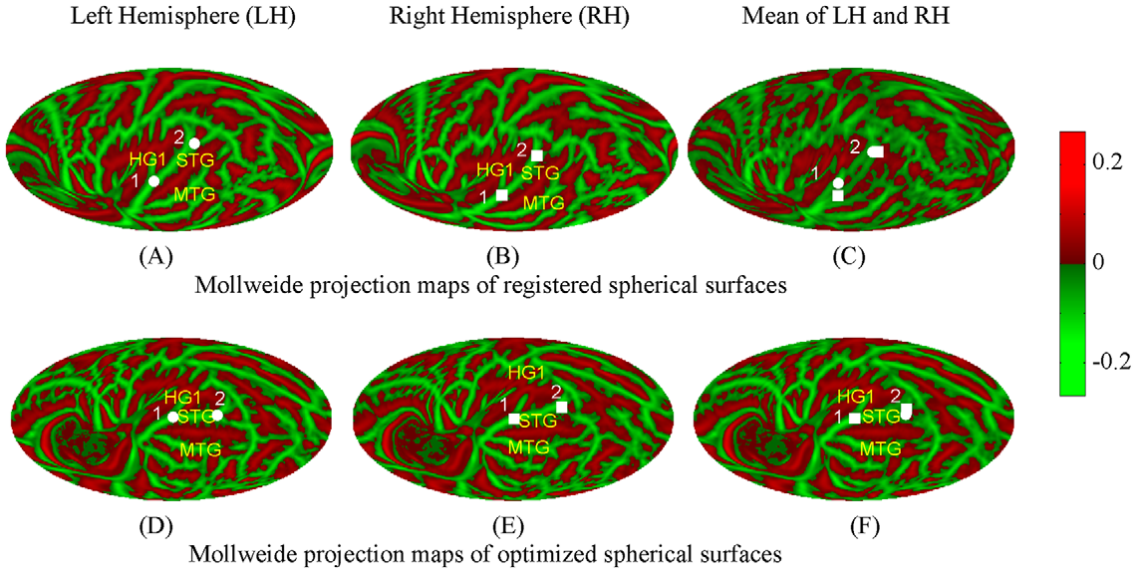
Mollweide projections of the mean anatomical maps across 138 subjects. (A) and (B) show the curvature patterns for the left hemisphere (LH) and mirrored right hemisphere (RH), respectively. Gyri  =  green, sulci  =  red. HG1  =  anterior Heschl's gyrus; MTG  =  middle temporal gyrus; STG  =  superior temporal gyrus. (C) is the average of LH and RH after the RH was mirrored and projected onto the LH. The anatomical structures are blurred and the two correspond landmarks 1 and 2 are dispersed on the average map (C) since the maps of LH and RH are not quite aligned. Two fiducial points used to crudely align the LH and RH, the intersection of HG1 and STG and intersection of STG and MTG, are shown as white circles (LH) and white squares (RH) in (D) and (E) for the LH and RH, respectively. The second row shows the projection maps in the proposed coordinate system in which fiducial point 1 is at the origin and fiducial point 2 is on the Equator, and the difference between the maps of LH and RH are aligned by numerical minimization. The gyral and sulcal structures are more clearly shown on the average map of LH and RH (F).

### Mollweide Projection

The visualization of the anatomical properties of the entire cortical surface of a hemisphere is complicated by the problem of displaying a convoluted three-dimensional surface in two dimensions. Researchers have introduced various flattened representations of the cortical surface to visualize its complex 3D structure in two dimensions [34]–[38]. Given that Freesurfer represents the cortical surface as a sphere, it is also possible to utilize standard cartographic projections to visualize cortical surface data on maps [39], such as the commonly used Mollweide equal-area projection.

**Figure 3:**
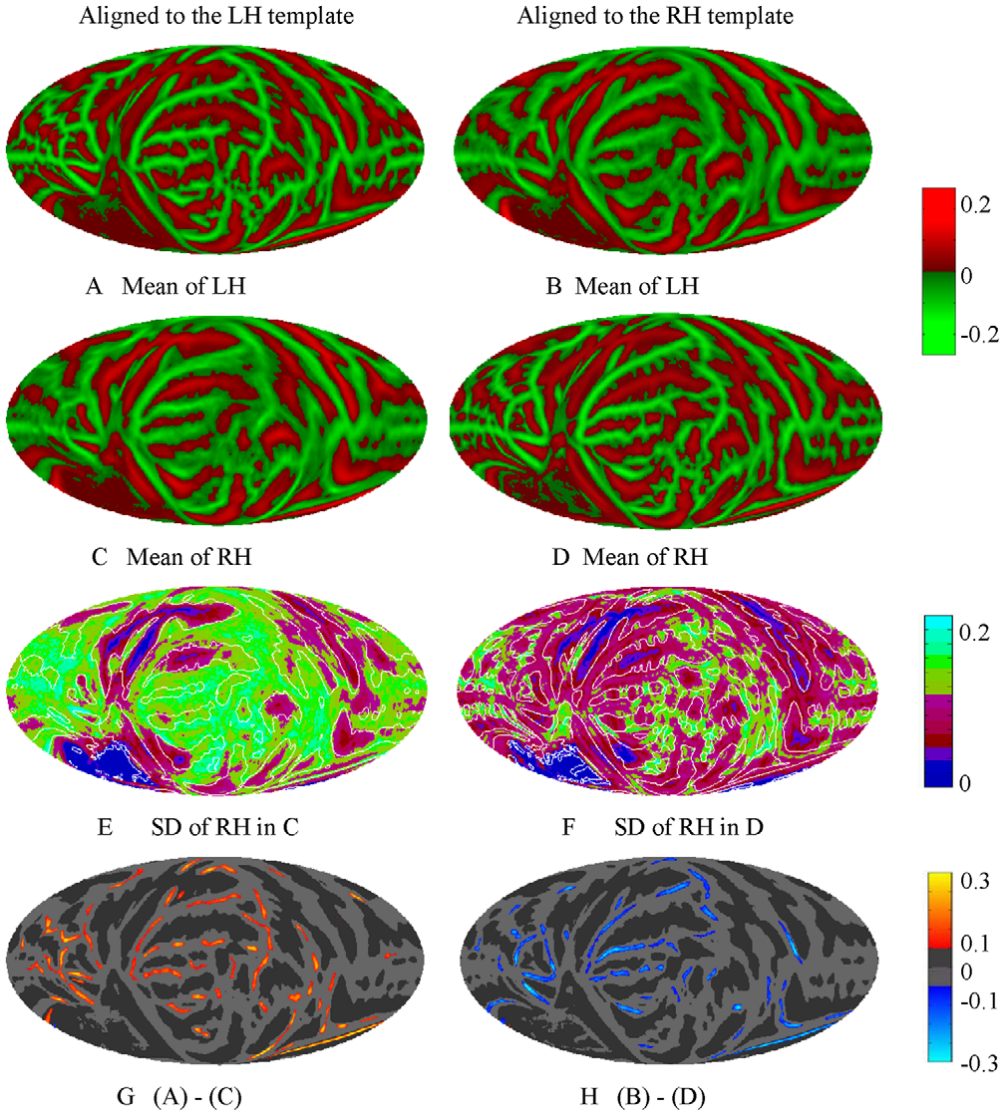
Mollweide projection maps of mean LH (A, B) and RH (C, D) across 138 subjects when they were coregistered to the LH template (A, C) and RH template (right B, D) by FreeSurfer, respectively. The mean curvature maps in B and C are blurrier then A and D, respectively, when the hemispheres were coregistered to the opposite hemisphere template. E and F show the standard deviation (SD) of RH when it is coregistered to the LH and RH templates, respectively. There is an overall jump in across-subject curvature variance of 74% in subjects' RH when coregistered to the LH template. The curvature difference calculation between LH and RH of the same group of 138 subjects shows LH has higher curvature values than RH (G) if LH and RH were aligned to the LH template, while LH has lower curvature value than RH (H) if both aligned to RH template.

The Mollweide equal-area projection [40], [41] is a pseudo-cylindrical projection of elliptical shape with minimal shape distortion in non-boundary regions (http://www2.ocgy.ubc.ca/~rich/map.html) in which the equator is represented as a straight horizontal line perpendicular to a central meridian, one-half its length. The Mollweide projection has the following useful features: (1) It is an equal-area projection, which means that two regions of equal area on the Mollweide also have equal area on the spherical map; (2) The scale is constant along any parallel, which are horizontal lines, and between parallels equidistant from the equator; (3) Mollweide projections are relatively simple to compute as they utilize only basic trigonometric functions; (4) In comparison with other equal area symmetrical projections, the Mollweide projection has low shape distortion, especially in central regions [42]; (5) The meridian-diameter circle in the center of the Mollweide ellipse represents one-half of a sphere's surface, but with much less perspective distortion than that of a simple 3D lateral view of a sphere.

**Figure 4:**
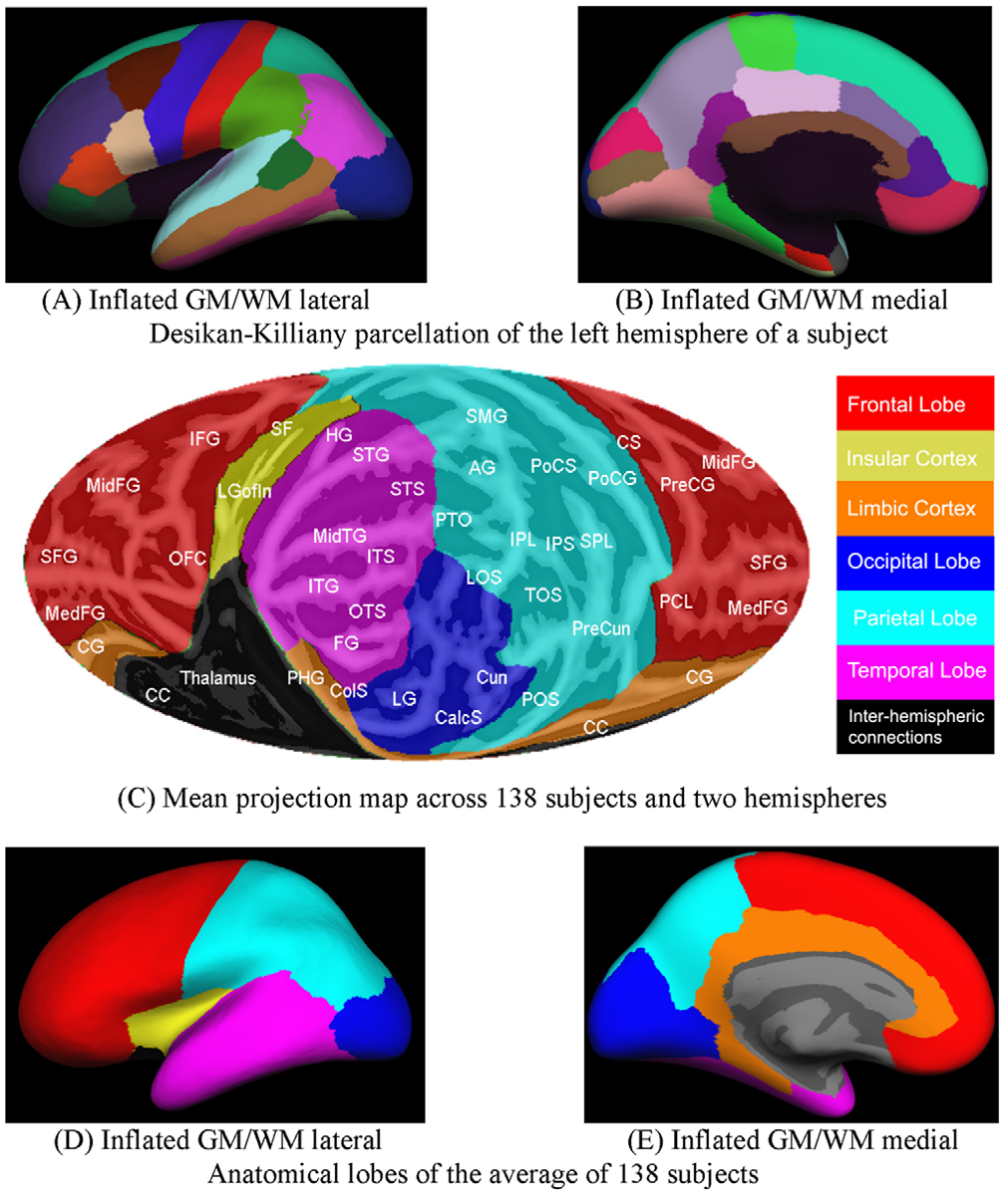
Desikan-Killiany [**45**] parcellation of LH cortex of one subject displayed on the lateral (A) and medial (B) sides of the inflated GM/WM boundary. (C) Mollweide projection map of the mean spherical cortical surface averaged across 138 subjects and two hemispheres. The sphere was rotated to position the temporal and occipital lobes in the front/central area of the Mollweide projection. Six anatomical areas were obtained based on the parcellation. FL: Frontal Lobe; IC: Insular Cortex; IHC: Inter-Hemispheric Connection; LC: Limbic Cortex; OL: Occipital Lobe; PL: Parietal Lobe; TL: Temporal Lobe. Anatomical structures (white labels): AG, angular gyrus; CC, corpus callosum; CG, cingulate gyrus; CalcS, calcarine sulcus; ColS, collateral sulcus; Cun, cuneate; CS, central sulcus; FG, fusiform gyrus; HG, Heschl's gyrus, IFG, inferior frontal gyrus; IPL, inferior parietal lobule; IPS, intraparietal sulcus; ITG, inferior temporal gyrus; ITS, inferior temporal sulcus; LG, lingual gyrus; LGofin, long gyrus of the insula; LOS, lateral occipital sulcus; MedFG, medial frontal gyrus; MidFG, mid-frontal gyrus, MidTG, middle temporal gyrus; PCL, paracentral lobule; PHG, parahippocampal gyrus; POS, parieto-occipital sulcus; PoCG, postcentral gyrus, PoCS, postcentral sulcus; PreCG, precentral gyrus; PreCun, precuneus; PTO, parietal/temporal/occipital junction; OTS, occipital temporal sulcus; SF, Sylvian fissure; SFG, superior frontal gyrus; SMG, supramarginal gyrus; SPL, superior parietal lobule; STG, superior temporal gyrus; STS superior temporal sulcus; TOS, transverse occipital sulcus. The lobes are also shown on the lateral (D) and medial (E) cortical surfaces.

**Table 1:** List of Desikan-Killiany parcellations for anatomical lobes.

Anatomical Lobes	Desikan-Killiany Parcellation
Frontal Lobe (FL)	caudal middle frontal cortex, frontal pole, lateral orbito frontal cortex, medial orbito frontal cortex, paracentral cortex, pars opercularis, pars orbitalis, pars triangularis, precentral cortex, rostral middle frontal cortex, superior frontal cortex
Insular Cortex (IC)	insular cortex
Limbic Cortex (LC)	caudal anterior cingulate cortex, isthmus cingulate cortex, parahippocampal gyrus, posterior cingulate cortex, rostral anterior cingulate cortex
Occipital Lobe (OL)	cuneus, lateral occipital cortex, lingual gyrus, pericalcarine cortex
Parietal Lobe (PL)	inferior parietal cortex, postcentral gyrus, precuneus cortex, superior parietal cortex, supramarginal gyrus
Temporal Lobe (TL)	posterior banks of the sts, entorhinal cortex, fusiform, inferior temporal gyrus, middle temporal gyrus, superior temporal gyrus, temporal gyrus pole, transverse temporal gyrus

Figure 1D shows the Mollweide projection of the cortical surface in a single subject, with the insula located at the center of the ellipse. The gyral structures within and surrounding the insula are displayed with minimal shape distortion and can be seen more clearly than on the 3D inflated representations. The principal disadvantage of the Mollweide projection are the discontinuities and shape distortions that occur in the four diagonal “corners” of the ellipse (i.e. distant from both the equator and the central meridian). For example, when the Mollweide is centered on the insula, the shapes of frontal and parietal gyri are distorted near the ellipse boundary. However, by positioning the region of greatest interest at the center of the Mollweide projection it is possible to visualize any cortical region with minimal shape distortion while preserving equal area cortical surface representation [40], [41].

**Figure 5:**
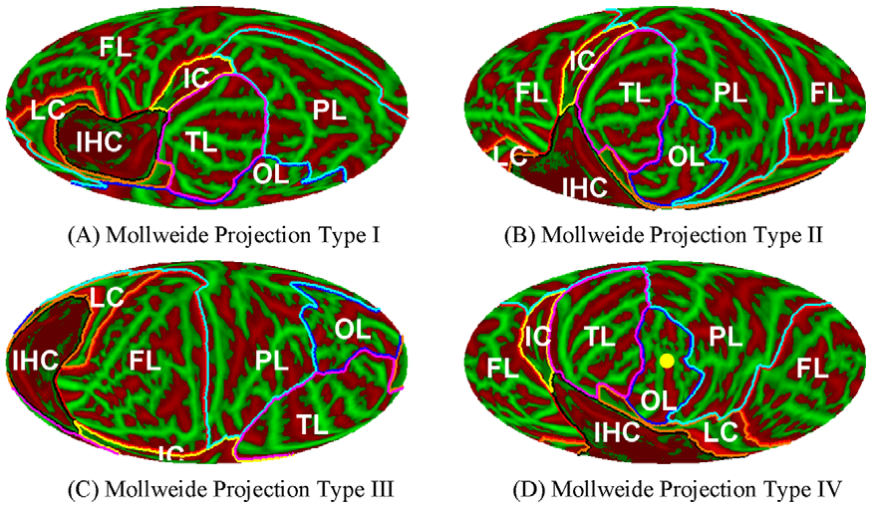
Different orientations of the Mollweide projection maps can be used to minimize shape distortion in primary regions of interest. (A) Type I projections minimize distortion of the insula and auditory cortex. (B) Type II projections permit the concurrent visualization of temporal, occipital and parietal cortex. (C) Type III projections minimize distortion in the frontal and parietal lobes. And (D) Type IV projections minimize shape distortions of visual regions surrounding the occipital pole (yellow spot). See Figure 4 for anatomical lobe labels.

The Mollweide projection is preferable to conformal projections such as the polar one proposed in Wandell et al. [39] and Sultan et al. [43] because conformal mappings have severe areal distortions that can force small pieces of cortex to take up large chunks of map space. In contrast, area-preserving projections allow visual comparisons of extents of parcellations across the contiguous cortical surface. The Mollweide projection also has good shape preservation throughout much of the central portion of the map. If desired, further reductions in the angular distortions at high latitude boundary areas can be obtained by switching to a more rectangular Tobler hyperelliptic projection [44] at a cost of additional mapping complexity, or by using projections such as the Eckert IV or Wagner VI [41], [42] that relax the equal-area property of the Mollweide near the poles.

**Figure 6:**
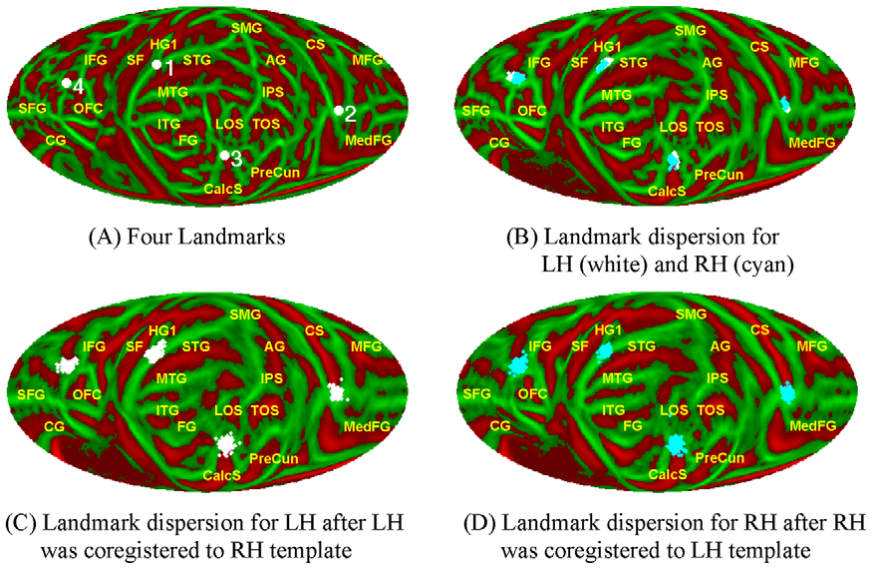
Manual landmark locations and dispersion. (A) Four landmarks on a sphere: (1) The intersection of the superior temporal gyrus and anterior Heschl's gyrus; (2) The superior vertex of the central sulcus; (3) The occipital pole; and (4) The inferior vertex of medial frontal sulcus (MedFS). (B) Landmarks on the mean map identified by one rater for the 138 subjects. White dots are used for LH landmarks and cyan dots for RH landmarks. The same landmarks have larger dispersion when the LH was coregistered to the RH template (C) and when the RHs were coregistered to LH template (D) by FreeSurfer. See Figure 4 for anatomical labels.

A two-dimensional (2D) common grid coordinate system was defined on the Mollweide projection map of the unified sphere by aligning LH and RH (see next section). All the anatomical properties, e.g., surface curvature, cortical thickness, surface area, convexity, sulcal depth, etc., of all the subjects were extracted and resampled from their own spherical surface into this common coordinate system. Then the analyses of all the anatomical properties were performed in this 2D common coordinate system on the Mollweide map.

**Table 2:** Dispersion radii (mm) of four landmarks of 138 subjects.

Landmarks	LH	LH to RH	RH	RH to LH	LR
1	4.5	8.8	3.2	6.0	4.5
2	2.2	6.4	2.3	6.9	4.5
3	2.9	6.2	2.8	5.9	3.8
4	3.5	6.9	3.8	7.3	4.6
Mean	3.3	7.1	3.0	6.5	4.4

(Four landmarks: (1) The intersection of STG and HG1; (2) Posterior vertex of CS; (3) Occipital pole; and (4) Inferior vertex of MedFS. LH: Left Hemisphere. RH: Right Hemisphere. LH to RH: LH coregistered to the RH template by FreeSurfer. RH to LH: RH coregistered to the LH template. LR: mean of LH and RH by our method).

### Alignment of the LH and RH

Although FreeSurfer inflation of the two hemispheres produces spherical surfaces on a normalized sphere [1], the co-registered spherical surface curvature maps for LH and mirror-imaged RH are misaligned because they were aligned to separate templates that are not optimally aligned to each other. Figure 2C shows the resulting mean map of coregistered LH (A) and mirror-imaged RH (B) averaged across 138 subjects. The gyral and sulcal structures are blurred and the corresponding two fiducial points (defined in Figure 2) 1 and 2 are dispersed on the mean map (C) since the FreeSurfer coregistered LH and mirror-imaged RH are not optimally aligned.

**Figure 7:**
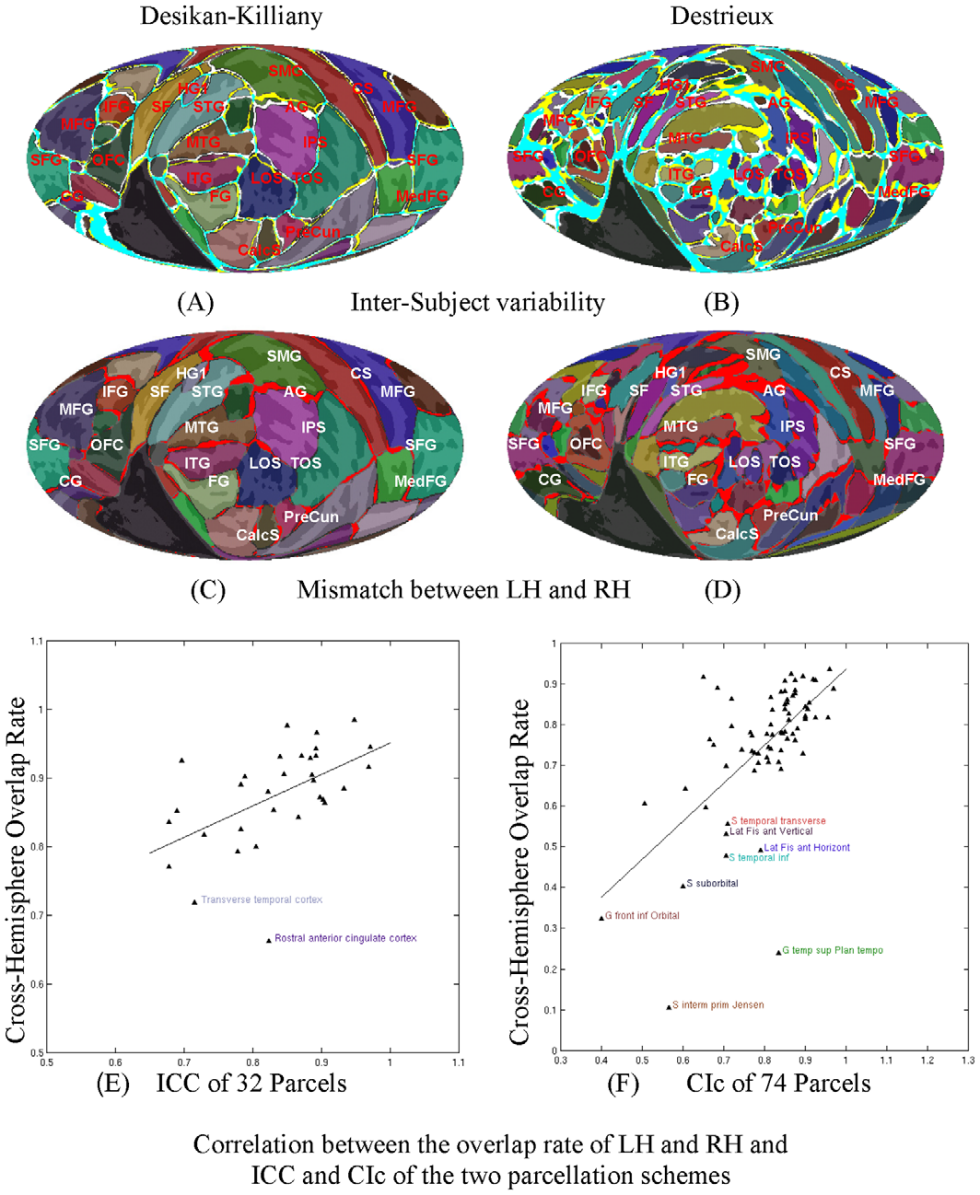
Inter-subject and inter-hemispheric variability of cortical parcellation. Semi-transparent color schemes show the parcellations defined in Desikan et al. [45] and Destrieux et al. [46]. (A) and (B) show the inter-subject variability of the two parcellation schemes across 138 subjects. Gyral and sulcal structures are shown by the light and dark gray in the background. Locations that are variably labeled in LH and RH are shown in white and yellow respectively, with cyan showing common variable areas. The bright red area in (C) and (D) shows the mismatch between LH and RH parcellation on the unified map. (E) and (F) show the scatter plots of the cross-hemisphere overlap rate of the two parcellation schemes for LH and RH (y axes) and the indices indicating agreement between automated and expert manual parcellation (x axes). Several anatomical labels with low overlapping rate are shown. The anatomical labels, intraclass correlations (ICCs) and concordance indices (CIs) are taken from Desikan et al. [45] and Destrieux et al. [46].

**Table 3:** Mean overlap rate and area of the Destrieux parcels averaged in each lobe.

	Overlap rate			FS Area (cm^2^)		Most-likely Area (cm^2^)	
Lobe	LH	RH	LR	LH	RH	LH	RH
FL	0.93	0.93	0.88	306.06	306.15	305.31	305.51
IC	0.92	0.92	0.85	20.71	20.11	20.70	20.03
LC	0.91	0.93	0.88	41.30	40.92	40.87	40.60
OL	0.93	0.92	0.86	112.01	113.93	112.00	113.86
PL	0.93	0.92	0.86	224.34	229.52	224.24	229.36
TL	0.92	0.93	0.86	161.16	156.14	161.12	156.28

FL: frontal lobe; IC: insular cortex; LC: limbic cortex; OL: occipital lobe; PL: parietal lobe and TL: temporal lobe. FS area: from FreeSurfer parcellations; Most-likely Area: from maximum likelihood parcellations.

FreeSurfer also provides a contra-surface coregistration method to compare one hemisphere to the other after non-linearly aligning the hemisphere to the template of the other. However, as shown in Figure 3, the resulting contra-averaged curvature maps are blurred (B and C) compared to the averaged curvature maps (A and D) obtained when each hemisphere is coregistered to its own templates. The intersubject variance (E) of contra-coregistered RH (C) increased substantially compared to the variance (F) of normally coregistered RH (D). Moreover, interhemispheric comparison of the surface curvature based on contra-surface coregistration will produce conflicting results depending on which hemisphere is used as the alignment template. For example, when the left-hemisphere template is used, subjects appear to have greater gyral and sulcal curvature (G) in the left hemisphere, whereas when the right-hemisphere template is used opposite results (H) are obtained.

**Table 4:** Mean overlap rate, CIc [46], and area (cm^2^) of the Destrieux parcels across 138 subjects.

		Overlap Rate			CIc		FS Area		Plurality Vote Area	
Index	Parcel Name	LH	RH	LR	LH	RH	LH	RH	LH	RH
1	Fronto-marginal gyrus (of Wernicke) and sulcus	0.93	0.91	0.77	0.73	0.68	7.66	7.09	7.67	7.11
2	Inferior occipital gyrus (O3) and sulcus	0.90	0.83	0.67	0.75	0.56	11.58	9.95	11.33	10.49
3	Paracentral lobule and sulcus	0.93	0.93	0.82	0.84	0.85	9.99	8.97	10.11	9.02
4	Subcentral gyrus (central operculum) and sulci	0.92	0.91	0.79	0.77	0.78	11.05	9.12	10.95	8.78
5	Transverse frontopolar gyri and sulci	0.93	0.91	0.77	0.63	0.67	4.05	7.45	3.96	7.03
6	Anterior part of the cingulate gyrus and sulcus (ACC)	0.93	0.92	0.82	0.84	0.91	15.42	18.33	15.19	18.53
7	Middle-anterior part of the cingulate gyrus and sulcus (aMCC)	0.93	0.94	0.86	0.85	0.85	9.44	10.78	9.03	10.54
8	Middle-posterior part of the cingulate gyrus and sulcus (pMCC)	0.95	0.94	0.87	0.88	0.86	10.00	11.14	9.95	10.97
9	Posterior-dorsal part of the cingulate gyrus (dPCC)	0.90	0.89	0.79	0.84	0.79	4.19	3.84	4.20	3.87
10	Posterior-ventral part of the cingulate gyrus	0.74	0.80	0.66	0.70	0.85	1.61	2.12	1.63	2.11
11	Cuneus (O6)	0.95	0.95	0.81	0.85	0.83	12.78	13.82	12.55	13.83
12	Opercular part of the inferior frontal gyrus	0.93	0.92	0.84	0.83	0.78	9.79	8.75	9.74	8.78
13	Orbital part of the inferior frontal gyrus	0.84	0.86	0.53	0.31	0.49	1.90	2.25	1.86	2.09
14	Triangular part of the inferior frontal gyrus	0.91	0.92	0.84	0.81	0.76	7.65	7.66	7.45	7.34
15	Middle frontal gyrus (F2)	0.92	0.90	0.81	0.85	0.83	31.22	28.30	31.01	27.92
16	Superior frontal gyrus (F1)	0.95	0.94	0.93	0.90	0.90	51.36	47.06	51.59	47.40
17	Long insular gyrus and central sulcus of the insula	0.84	0.87	0.74	0.78	0.79	3.00	3.28	2.98	3.28
18	Short insular gyri	0.89	0.89	0.85	0.75	0.79	4.22	3.99	4.28	3.96
19	Middle occipital gyrus (O2, lateral occipital gyrus)	0.89	0.89	0.76	0.77	0.77	14.69	15.78	14.59	15.71
20	Superior occipital gyrus (O1)	0.94	0.92	0.82	0.76	0.68	11.26	13.48	11.42	13.35
21	Lateral occipito-temporal gyrus (fusiform gyrus, O4-T4)	0.94	0.93	0.88	0.85	0.85	12.71	11.72	12.49	11.34
22	Lingual gyrus, lingual part of the medial occipito-temporal gyrus, (O5)	0.95	0.94	0.82	0.90	0.84	21.04	20.04	21.13	20.17
23	Parahippocampal gyrus,	0.90	0.91	0.86	0.92	0.89	10.10	10.33	10.37	10.51
24	Orbital gyri	0.94	0.93	0.86	0.86	0.85	18.15	18.71	18.16	18.70
25	Angular gyrus	0.91	0.88	0.72	0.82	0.82	16.50	20.59	16.72	20.26
26	Supramarginal gyrus	0.94	0.92	0.79	0.83	0.79	20.95	18.87	20.96	19.21
27	Superior parietal lobule (lateral part of P1)	0.93	0.91	0.81	0.81	0.80	20.33	16.40	20.06	16.74
28	Postcentral gyrus	0.94	0.95	0.88	0.89	0.91	15.14	13.83	15.16	13.69
29	Precentral gyrus	0.94	0.94	0.87	0.91	0.91	17.80	17.60	17.63	17.58
30	Precuneus (medial part of P1)	0.92	0.92	0.84	0.86	0.84	17.49	17.17	17.59	17.42
31	Straight gyrus, Gyrus rectus	0.93	0.91	0.86	0.84	0.84	7.25	5.44	7.25	5.52
32	Subcallosal area, subcallosal gyrus	0.47	0.60	0.51	0.60	0.61	1.19	1.01	0.37	0.75
33	Anterior transverse temporal gyrus (of Heschl)	0.93	0.92	0.82	0.83	0.79	3.61	2.57	3.58	2.52
34	Lateral aspect of the superior temporal gyrus	0.94	0.95	0.79	0.90	0.89	14.28	12.19	14.40	12.16
35	Planum polare of the superior temporal gyrus	0.84	0.87	0.82	0.71	0.82	4.33	4.90	4.17	4.69
36	Planum temporale or temporal plane of the superior temporal gyrus	0.92	0.91	0.62	0.85	0.82	7.58	5.91	7.51	5.86
37	Inferior temporal gyrus (T3)	0.91	0.93	0.81	0.81	0.81	18.76	17.26	18.79	16.62
38	Middle temporal gyrus (T2)	0.93	0.95	0.82	0.84	0.88	19.76	21.04	19.52	20.84
39	Horizontal ramus of the anterior segment of the lateral sulcus (or fissure)	0.89	0.88	0.64	0.71	0.87	2.18	2.64	2.11	2.73
40	Vertical ramus of the anterior segment of the lateral sulcus (or fissure)	0.75	0.82	0.81	0.70	0.71	2.35	1.68	2.63	1.71
41	Posterior ramus (or segment) of the lateral sulcus (or fissure)	0.95	0.95	0.83	0.93	0.82	8.41	9.55	8.37	9.54
42	Medial Wall	0.97	0.98	0.97			21.07	21.72	21.72	22.00
43	Occipital pole	0.94	0.93	0.91	0.70	0.67	15.93	23.36	15.93	23.27
44	Temporal pole	0.94	0.96	0.96	0.85	0.85	11.36	11.61	11.52	11.60
45	Calcarine sulcus	0.95	0.94	0.94	0.94	0.91	17.53	17.04	17.64	17.22
46	Central sulcus (Rolandos fissure)	0.97	0.97	0.98	0.97	0.97	23.23	22.09	23.29	22.24
47	Marginal branch (or part) of the cingulate sulcus	0.96	0.96	0.97	0.92	0.87	7.89	9.30	7.87	9.25
48	Anterior segment of the circular sulcus of the insula	0.94	0.92	0.92	0.82	0.81	3.69	4.49	3.68	4.56
49	Inferior segment of the circular sulcus of the insula	0.94	0.92	0.94	0.87	0.84	10.45	9.09	10.59	9.34
50	Superior segment of the circular sulcus of the insula	0.94	0.94	0.96	0.83	0.84	12.25	9.66	12.24	9.69
51	Anterior transverse collateral sulcus0.87	0.90	0.93	0.92	0.84	0.87	7.03	7.03	7.31	7.10
52	Posterior transverse collateral sulcus0.64	0.88	0.80	0.84	0.69	0.64	3.15	4.24	3.14	4.49
53	Inferior frontal sulcus	0.94	0.92	0.92	0.86	0.77	16.28	14.90	16.06	15.09
54	Middle frontal sulcus	0.89	0.89	0.89	0.67	0.77	11.89	15.48	12.03	15.70
55	Superior frontal sulcus	0.93	0.94	0.94	0.83	0.87	21.04	18.96	20.83	18.53
56	Sulcus intermedius primus (of Jensen)	0.86	0.66	0.39	0.58	0.55	2.98	3.28	2.74	2.87
57	Intraparietal sulcus (interparietal sulcus) and transverse parietal sulci	0.93	0.92	0.88	0.85	0.79	22.70	23.31	22.58	23.00
58	Middle occipital sulcus and lunatus sulcus	0.92	0.91	0.94	0.88	0.84	8.67	7.68	8.83	7.72
59	Superior occipital sulcus and transverse occipital sulcus	0.94	0.94	0.96	0.87	0.88	9.46	11.45	9.55	11.62
60	Anterior occipital sulcus and preoccipital notch (temporo-occipital incisure)	0.79	0.86	0.75	0.51	0.50	5.97	5.28	6.45	5.19
61	Lateral occipito-temporal sulcus0.77	0.88	0.89	0.91	0.72	0.77	6.52	6.75	6.57	6.71
62	Medial occipito-temporal sulcus (collateral sulcus) and lingual sulcus	0.95	0.96	0.97	0.90	0.90	14.64	14.22	14.25	13.90
63	Lateral orbital sulcus	0.90	0.83	0.90	0.72	0.63	3.17	3.29	3.29	3.36
64	Medial orbital sulcus (olfactory sulcus)0.96	0.94	0.93	0.96	0.95	0.96	4.78	4.84	4.67	4.83
65	Orbital sulci (H-shaped sulci) I,	0.95	0.95	0.97	0.96	0.96	9.85	10.49	9.84	10.53
66	Parieto-occipital sulcus (or fissure)	0.96	0.95	0.97	0.95	0.90	14.19	14.90	14.27	14.86
67	Pericallosal sulcus (S of corpus callosum)	0.86	0.91	0.91	0.86	0.94	6.41	8.21	6.52	8.08
68	Postcentral sulcus	0.94	0.94	0.96	0.89	0.87	20.69	17.92	20.96	17.98
69	Inferior part of the precentral sulcus	0.94	0.94	0.95	0.85	0.88	11.38	12.16	11.40	12.10
70	Superior part of the precentral sulcus	0.94	0.94	0.96	0.83	0.85	10.88	11.43	10.88	11.38
71	Suborbital sulcus (sulcus rostrales, supraorbital sulcus)	0.72	0.71	0.75	0.60	0.60	4.48	2.68	4.53	2.85
72	Subparietal sulcus	0.92	0.92	0.95	0.91	0.84	7.73	8.90	7.67	8.68
73	Inferior temporal sulcus	0.77	0.81	0.85	0.69	0.72	10.82	9.80	10.27	10.14
74	Superior temporal sulcus (parallel sulcus)	0.96	0.95	0.97	0.93	0.91	38.63	42.81	38.66	43.41
75	Transverse temporal sulcus	0.88	0.88	0.91	0.70	0.72	2.57	2.18	2.58	2.21

For LH and RH the overlap rate ( = common area/mean area) was computed comparing individual subject hemispheres to the aggregate plurality vote map in the same hemisphere whereas LR was computed by comparing each hemisphere to the opposite hemisphere's plurality vote map. FS area: from FreeSurfer parcellations.

We therefore developed an unbiased procedure for aligning the anatomy of the two hemispheres using the following steps:

**Figure 8:**
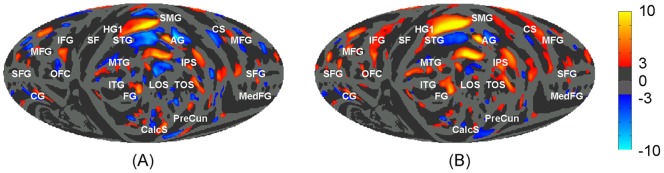
Mollweide projection maps of the mean difference of the convexity (A, in mm) and sulcal depth (B, in mm, defined in Van Essen [**2**]) between LH and RH. The difference was averaged across 138 subjects. Positive values are left > right. See Figure 4 for anatomical labels.

1. High resolution anatomical images of all subjects were segmented, inflated and coregistered to the spherical coordinate system by FreeSurfer. The LH and RH surfaces were averaged across the 138 subjects using FreeSurfer.
2. A grid coordinate space was defined on the sphere. The averaged LH an RH were resampled in the grid space. The RH was mirrored in longitudinal direction.
3. Two consistent and easily identified anatomical landmarks within the lateral temporal cortex [2], intersection of STG and HG1 and bifurcation point on STG, as shown in Figure 2, were identified on the averaged LH and mirrored RH. Fiducial point 1 was transferred to the center of the sphere and fiducial point 2 on the Equator by spherical translation and rotation on the averaged LH and mirrored RH. This step brought the hemispheres into coarse alignment.
4. The averaged LH and mirrored RH were optimally aligned by minimizing the global root-mean-square difference (RMSD) in curvature between the hemispheres over a search space of ±10 mm in spherical translation and a ±20° in rotation of around the north pole. We found that the optimal alignment of the LH and mirrored RH occurred following a translation of 1.1 mm in latitudinal direction, 1 mm in longitudinal direction, and a rotation of 11.8° around the north pole.
5. The resulting total rigid-body spherical transformation, including coarse alignment and optimization alignment, was then applied to the coregistered LH and RH of each subject. The mean of LH and mirrored RH, i.e. the hemispherically-unified common template space, can be obtained from LH and RH of all subjects after they were transferred and resampled into the grid space.

**Table 5:** Comparison of mean sulcal depth, absolute cortical surface curvature, bending energy, cortical surface area, and mean cortical thickness across hemispheres for 138 right-handed subjects (LH: Left Hemisphere; RH: Right Hemisphere; WH: Whole Hemisphere; FL: frontal lobe; IC: insular cortex; LC: limbic cortex; OL: occipital lobe; PL: parietal lobe and TL: temporal lobe; interhemispheric comparisons significance labeled *P<0.01*, P<0.001, *P<0.0001*).

	Sulcal Depth (mm)		Absolute Curvature		Bending Energy (x100)		Area (cm^2^)		Thickness (mm)	
	LH	RH	LH	RH	LH	RH	LH	RH	LH	RH
WH	***7.89***	***7.08***	0.138	0.138	2.75	2.74	942.7	943.3	2.58	2.59
FL	***6.87***	***6.11***	***0.137***	***0.138***	***2.70***	***2.73***	331.5	331.4	2.71	2.71
IC	***24.5***	***22.8***	***0.110***	***0.113***	***2.08***	***2.18***	***22.8***	***21.8***	3.13	3.15
LC	4.68	4.95	**0.133**	**0.135**	2.67	2.70	**45.3**	**44.1**	*2.77*	*2.73*
OL	**6.03**	**5.62**	0.148	0.148	3.01	3.02	*122.0*	*124.1*	2.06	2.07
PL	***8.95***	***8.10***	***0.137***	***0.136***	***2.71***	***2.68***	***245.6***	***251.4***	2.38	2.39
TL	***8.26***	***7.03***	***0.138***	***0.136***	***2.81***	***2.74***	***175.5***	***170.5***	***2.85***	***2.91***

The spherical rigid-body transformation used in the above procedure preserves the locations of gyral and sulcal structures of LH and RH of all subjects in their original coordinate systems as well as mapping them into hemispherically-unified coordinate space. This fixed, omnibus transformation of the two hemispheres into a common space compensates for some of the asymmetries in the cerebral hemispheres associated with Yakovlevian torque and petalia [27]–[30]. Fig. 2D**–**F show the mean Mollweide maps across all the subjects for LH, RH and the average of LH and RH, respectively, after the spherical maps of all subjects were aligned prior to Mollweide projection.

**Figure 9:**
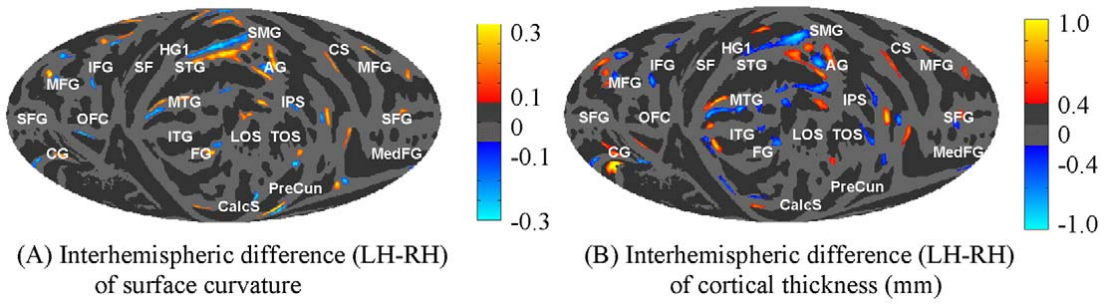
Interhemispheric differences (*P*<0.005) of mean cortical surface curvature (A) and mean cortical thickness (in mm) (B) across 138 subjects on the mean Mollweide projection maps Type II. See Figure 4 for anatomical labels.

### Cortical Parcellation

FreeSurfer also provides neuroanatomical parcellation of the cortex, coarser parcellations as defined by Desikan-Killiany [45] and finer parcellations as defined by Destrieux [46]. Figure 4A and B show Desikan-Killiany parcellations on the inflated gray/white matter (GM/WM) boundary. The parcellation boundaries were defined within each hemisphere by plurality vote after overlapping the parcellations of the 138 subjects. This permitted an examination of inter-subject variability in anatomical structure by quantifying the intersubject variance in the size and location of Desikan-Killiany and Destrieux parcels within each hemisphere. A similar plurality vote procedure was used to define parcellations in hemispherically unified coordinates. In order to calculate and compare the properties in each anatomical lobe, the following anatomical areas were identified based on the Desikan-Killiany parcellation (See Table 1 for details): the frontal lobe (FL), insular cortex (IC), limbic cortex (LC), occipital lobe (OL), parietal lobe (PL) and temporal lobe (TL). Figure 4C shows the mean Mollweide projection map of the frontal, temporal, parietal and occipital lobes averaged across 138 subjects and two hemispheres. The temporal and occipital lobes were positioned so that the auditory and visual cortex can be viewed simultaneously. The approximate locations of selected anatomical landmarks are labeled. The anatomical lobes as projected onto the partially inflated cortical surface are shown in Figure 4D and 4E.

As mentioned earlier, the anatomical region of greatest interest should be projected at the center of the map because Mollweide projections have minimal shape distortion in the central regions of the projection. Figure 5 shows four different Mollweide projections centered on the different regions of interest: the superior temporal plane to examine auditory cortex (Type I, 5A), the inferior temporal lobe to visualize temporal, occipital and parietal cortex (Type II, 5B), the frontal and parietal lobes (Type III, 5C), and visual cortex surrounding the occipital pole (Type IV, 5D). Type II projections are used throughout this report. The conversion between MNI space, Talairach space and Mollweide map coordinates can be performed by the Flat-Mapper at http://www.ebire.org/hcnlab/cortical-mapping/.

### Accuracy of the Alignment of LH and RH

The averaged Mollweide map was generated by minimizing the difference of the surface curvature between the LH and RH. We checked the success that our cross-hemisphere registration in several ways, described in detail below.

First we evaluated the landmark dispersion on normally coregistered LH, RH, the averaged map, and contra-coregistered LH and RH. Two raters selected the following four landmarks (shown in Figure 6A) in four anatomical lobes on normally coregistered LH and RH for 138 subjects: (1) The intersection of STG and HG1 in TL; (2) Posterior vertex of CS in PL; (4) Occipital pole in OL; and (5) Inferior vertex of medial frontal sulcus (MedFS) in FL. The dispersion radius is the great circle distance on the sphere between one landmark and the center of mass of the same landmarks from all subjects. The dispersions of landmarks on LH and RH, normally or contra coregistered, reflect the accuracy by FreeSurfer's spherical co-registration, while the dispersions on LR, the mean of LH and RH, reflect the accuracy of our alignment method of LH and RH.

The second set of checks evaluated how accurately FreeSurfer-defined parcellations were mapped to corresponding locations in the hemispherically-unified space. Maps reflecting interhemispheric mismatches for the Desikan-Killiany and Destrieux parcellations were computed to examine the accuracy of interhemispheric alignment based on the assumption that similar cortical regions in each hemisphere should be co-located in hemispherically-unified coordinate space. As stated in the previous section and shown in Figure 4 A–C, the parcellation boundaries in the common space were defined by plurality vote after overlapping the parcellations of the 138 in both hemispheres, and the anatomical lobes were defined from the parcellations in the common space. Thus it is necessary to examine the accuracy of interhemispheric alignment of all parcels. The mean cross-hemisphere overlap rate of each parcel was used to evaluate the alignment of LH and RH parcellations and visualize areas of misalignment. The success in matching corresponding parcels across hemispheres was compared with the accuracy of FreeSurfer's automated parcellations when checked against expert-manual parcellation [45], [46]. In particular, we wanted to confirm that accurately identified anatomical parcellations within each hemisphere were also accurately collocated across hemispheres.

The third set of checks analyzed curvature and sulcal depth measures to evaluate overall interhemispheric alignment in unified space. The accuracy of continuous sulcal and gyral alignment across the two hemispheres was also estimated by generating difference maps between LH and RH convexity [33] from FreeSurfer and differences in sulcal depth [2] using Caret v 5.51. Convexity is the distance a surface point moved during surface inflation, while the sulcal depth is the distance from the cortical surface to the envelope surface of the cortex.

Finally, we examined the quality of cross-hemispheric alignment with respect to the quality of FreeSurfer's intersubject alignment by examining the amount of overall cortical surface bending energy, the square of mean curvature after subtracting overall average mean curvature [47]. Bending energy better reflects the number of gyri and sulci in a region. Thus, reduction in bending energy in average maps relative to individual maps reflects the degree of misalignment of gyral and sulcal details of each individual hemisphere. A comparison of bending energy in the hemispherically-unified average map relative to average LH and RH maps reflects the degree to which the unified coordinate system accurately reflected the average gyral and sulcal curvature of each individual hemisphere.

### Hemispheric Asymmetries

Hemispherically-unified map provide a common space to compare the hemispheric asymmetries of anatomical features. We used the unified interhemispheric spherical map and its Mollweide projection to statistically evaluate the accuracy of co-registration within each hemisphere and the effects of hemisphere, gender and cortical lobe on the surface area, convexity, sulcal depth, bending energy, absolute curvature and thickness [48] of the cortex. Further linear regressions were used to determine if significant effects could be accounted for using covariates such as age and total cortical surface area.

## Results

### Landmark Dispersion

Figure 6B shows the four landmarks identified by one rater for LH (white dots) and RH (cyan dots) of the 138 subjects on the hemispherically-unified map. The landmark groups overlapped relatively well on the mean map. The same landmarks becomes more dispersed on the contra-coregistered LH (Figure 6C) and RH (Figure 6D). Table 2 shows the dispersion radii of all landmarks on normally coregistered LH, RH, the aligned LH and RH mean, and the contra-coregistered LH and RH. The mean dispersion radii were 3.3 mm in the LH and 3.0 mm in the RH, but they increased by 33% and 46%, respectively, when landmarks were aligned using the hemispherically-unified coordinate system. In contrast, the dispersions increased by 115% and 117%, respectively, when the hemispheres were co-registered to the contralateral hemispherical templates.

### Intersubject Variability of Cortical Surface Structure

Figure 7A shows the intersubject variability of parcellation assignment in Desikan-Killiany parcellation in both hemispheres (cyan), regions with additional variability in the LH (white), and RH (yellow) alignment alone. Most parcellation units showed good agreement across subjects in both the left and right hemispheres. Figure 7B shows corresponding intersubject variability measurements for the smaller Destrieux parcels. Regions of increased intersubject anatomical variability were found in the inferior temporal lobe, the superior temporal plane and posterior Sylvian fissure near the temporal/parietal junction, as well as in the anterior mid- and superior frontal gyri. In contrast, minimal intersubject variability was found around the central sulcus and the anterior superior temporal sulcus. The accuracy of within-hemisphere parcel assignment was similarly high in different cortical lobes as shown in Table 3. More detailed overlap rates, sizes, and hemispheric asymmetries of each Destrieux parcel are provided in Table 4.

### Cross-Hemisphere Alignment

Figure 7C and D show the regional misalignments between LH and RH on the hemispherically-unified map for the two FreeSurfer parcellation schemes. The accuracy of interhemispheric alignment was moderately reduced in peri-insular regions was generally high throughout the other cortical lobes including the frontal lobe. Regions with the greatest interhemispheric misalignment were concentrated around the Sylvian fissure and included the inferior frontal gyri, the circular sulcus of the insula, the transverse temporal gyri and sulci, the posterior superior temporal plane and posterior superior temporal gyrus, and the angular gyrus (Table 3 and 4).

In general, we found a strong positive correlation between the accuracy of within and across-hemisphere parcellation overlap and the accuracy of automated parcellation by Freesurfer, as shown in Figure 7E and F and in Table 4. Parcellations more accurately identified within hemispheres were usually aligned more accurately across hemispheres. However, several regions with accurate within-hemispheres alignment showed large hemispheric asymmetries including the posterior ramus of the Sylvian fissure, the planum temporale, the subcentral gyrus and sulcus, the superior portion of the circular gyrus and sulcus of the insula, and the angular gyrus.

Interhemispheric differences in convexity (Figure 8A) and sulcal depth (Figure 8B) show similar distributions for measurements made with these independent methods. The major asymmetries identified in both measurements were in the posterior Sylvian fissure and superior temporal sulcus. In general, the results agree well with the results of Im et al. [49] and Van Essen [2].

A final check on cross-hemisphere alignment is provided by the overall bending energy in individual subjects versus the bending energy in averaged LH, RH, and unified map. The bending energy is a square of the curvature and is therefore sensitive mainly to the presence of gyral crests and sulcal fundi [47]. The mean bending energy (x100) for individual hemispheres was 2.75 for the LH and 2.74 for the RH as shown in Table 5. Bending energy varied among different lobes, ranging from 2.13 in insular cortex to 3.01 in the occipital lobe. Differences in bending energy reflect the variations in the complexity of folding patterns in the different lobes, with the highest complexity found in occipital cortex. Fine details of individual anatomy were lost in population-average maps of the LH and RH. As a result, the bending energy of population average maps was reduced to 1.62 for the LH and 1.58 for the RH. This suggests that about 58% of the cortical surface features seen in individual brains were preserved in FreeSurfer averages. There was a small additional loss of anatomical detail in the hemispherically-unified average, with an associated bending energy of 1.38 (i.e., 50% preservation of surface curvature from the individual maps). This result suggests that the hemispherically unified map preserves approximately 86% of the anatomical features present in the average LH and RH maps derived from separate templates.

### Hemispheric Asymmetries in Cortical Anatomy

Table 5 also provides measurements of mean thickness, absolute curvature, sulcal depth, and surface area on the whole surface and in the six anatomical lobes of the LH and RH. Figure 9 shows the interhemispheric differences (*P*<0.005) of mean cortical surface curvature and mean cortical thickness (in mm) across 138 subjects. There were no global hemispheric asymmetries except in mean sulcal depth, where the LH showed significantly deeper sulci than the RH (mean 0.8 mm, p<0.0001). The hemispheric asymmetry in sulcal depth was found in all lobes except limbic cortex, and remained significant (0.8 mm LH > RH, t_270_  = 8.1, p<0.0001) for whole brain measures after performing a secondary linear regression with the additional covariates of age, gender, total area, and bending energy, none of which produced significant effects on their own. Small but significant lobe-specific interhemispheric asymmetries were also found in curvature (greater in the RH frontal lobe, insula, and greater in LH parietal and temporal lobes), bending energy (greater in the RH frontal lobe, insula, and in the LH parietal and temporal lobes), area (larger in the LH insula, limbic cortex, and temporal lobe and larger in the RH parietal lobe), and in cortical thickness (greater in the LH parietal lobe). Insular cortex was the most asymmetric lobe: its area was 4.5%, larger in the LH than the RH, and the LH insula was 1.7 mm deeper, while the RH insula contained 4.7% higher bending energy. Finally we note that the thickness measurements within various lobes are in good agreement with Fischl and Dale [48] and Salat et al. [50].

## Discussion

### Accuracy of Alignment of the Cortical Surface to Freesurfer Templates

Freesurfer parcellations of the cortical surface were accurately aligned across subjects. In particular, the gyral and sulcal structures of the frontal and parietal lobes were as accurately co-registered as other cortical regions. This indicates that cortical surface mapping techniques can be as usefully applied to studies of the topographic organization of high-level association cortex as to sensory cortex, where they have proven essential in revealing cortical field organization. Regions of high anatomical variability revealed with the automated method were generally similar to those identified manually by Ono et al. [51], namely in temporo-parietal areas and some frontal areas.

### Accuracy of the Alignment of LH and RH

The method proposed in this paper is to align the LH and RH by global minimization algorithm using rigid body spherical transformation. The accuracy of the method was examined by several ways. As shown in Figure 6, the landmark groups from normally coregistered LH and RH overlapped relatively well on the mean map. The reason is that our alignment method optimized the global difference using the rigid body transformation which preserves the local gyral and sulcal structure. Landmark dispersions increased somewhat after alignment to the hemispherically-unified coordinate system, but much less than after the alignment to the contralateral hemisphere. The improved precision of the hemispherically-unified coordinate system relative to contra-hemisphere alignment can also be observed from the maps of mean LH and RH curvature shown in Figure 5B which have more accurate gyral and sulcal structure and lower variance than the mean maps shown in Figure 3B and 3C from the contra-surface coregistration by FreeSurfer.

The mismatches of the parcellations in hemispherically unified space were predicted by the inter-subject within-hemisphere variability of the parcels for most regions as shown in Figure 7. However, some regions showed clear hemispheric asymmetries. These regions (e.g., the planum temporale) were well aligned within hemispheres by showed less precise cross-hemisphere alignment. Compared to the results of the contra-surface coregistration method shown in Figure 3, the interhemispheric differences of anatomical features, as shown in Figures 8 and 9, have a unique solution in the hemispherically unified coordinate space. In contrast, two different solutions would be needed if contra-hemisphere registration were used.

The optimum alignment parameters between the LH and RH were also tested using seven randomly chosen subsets containing 10, 20, 30, 50, 70, 90, and 120 subjects. The results show that the difference between the alignment parameters of all the subsets and the full group are at worst ± 0.1 mm for spherical translation and ± 0.2 degree for spherical rotation. Also, the parameters stabilize upon repeated subgroup sampling when group sizes exceeded 50 subjects.

We next used similar procedures to align the LHs and RHs of two groups of 50 young (mean age  = 21.9 years) and 50 older control subjects (mean age  = 66.6 years) from the public OASIS T1W database [52]. The optimum alignment parameters (translation in latitudinal and longitudinal directions and rotation angle) were [1.4 mm, 1.1 mm, 12.1°] and [1.4 mm, 1.1 mm, 12.3°] for the two groups, respectively. These results were very close to the results of [1.1 mm, 1.0 mm, 11.8°] of the main subject group: i.e., the maximal difference in realignment locations between all three groups was less than 2 mm for any point on the cortical surface.

### Hemispheric asymmetries

The hemispherically-unified FreeSurfer-based coordinate system introduced here permits objective inter-hemispheric comparisons of anatomical curvature, structural variability and cortical thickness. It also permits interhemispheric comparisons of other coregistered images including functional activations [8], [53]. The hemispherically-unified Mollweide maps preserve the accuracy of the normalized spherical systems implicit in the independent LH and RH FreeSurfer templates by means of fixed, rigid-body spherical transformations. By using the hemisphere-specific templates rather than using a single hemisphere's template for surface registration, we chose to privilege intrahemispheric co-registration accuracy over interhemispheric comparisons [28]–[30]. Nevertheless, the unified coordinate system preserves the large majority of major anatomical features present in the individual LH and RH average cortical surface representations produced by FreeSurfer.

While the asymmetries that we observed depend in large part on the asymmetries inherent in the LH and RH FreeSurfer templates, the asymmetries in the templates likely reflect true interhemispheric differences in structure [30]. Deforming each individual subject's inflated hemisphere to the opposite hemisphere's template [28], [29], [51], [54] results in a considerable loss in coregistration precision and a resulting increase in variance (Figure 3).

Alternatively, a hemispherically unbiased template can be developed [55] that would result in a smaller reduction in coregistration precision than an opposite hemisphere template, but that would likely increase alignment imprecision and tend to underestimate true hemisphere differences in anatomy. One final alternative would be to select a few dozen reliable anatomical landmarks and nonlinear deformation in order to map the LH and RH into a common space [56]. However such a mapping introduces subjectivity, requires anatomical expertise and would be quite time consuming for large data sets.

The unified coordinates can be used to reveal regions where LH and RH gyral and sulcal anatomy are distinct. Measures of area, sulcal depth, gyral curvature and cortical thickness all indicated that the major regions of reliable interhemispheric anatomical asymmetry were found in peri-Sylvian cortex, including the insula and posterior superior temporal plane. These areas form the core of language-related brain regions [57] and also show large differences in pericortical tissue properties [16].

When analyzing the anatomical dataset, we found no overall hemispheric asymmetries in cortical thickness, consistent with the results of Janauskaite et al. [58] and Salat et al. [50], but in contrast to previous reports of a general LH increase in thickness accompanied by regional thickness asymmetries exceeding 5% [31], [59]. In our data, only the temporal lobe had a significant thickness asymmetry (2%, Table 5).

Our temporal and occipital lobe hemispheric areal asymmetries agree with those in Lyttelton et al. [55], whereas the limbic cortex asymmetry does not. The parietal lobe asymmetry in area is also partly in conflict with Lyttelton et al. [55] – in particular the strong LH > RH area asymmetry of the postcentral gyrus reported by Lyttelton et al. [55] was absent in our data. There have also been reports of RH > LH volume asymmetries in the superior parietal cortex in postmortem data [60], but those were also not evident in our results. However, the LH > RH asymmetry in limbic cortex area, particularly in the anterior cingulate (Figure 9A and B), agrees with a volume asymmetry reported by Huster et al. [61]. We also found systematic differences in thickness between gyri and sulci that are in close agreement with previously reported values [48], [50], [62]–[64].

### Limitations

The method in this paper uses the surface curvature of all the subjects to align the LH and RH and generate a unified template for hemispheric analysis. However, the optimized alignment parameters that we found for our right-handed subject groups may not be optimal for the study of other subject groups (e.g., left-handed subjects, etc.). Nevertheless, we verified that the optimal alignment parameters obtained in our subject group were very similar to those obtained from two right-handed subject groups of different ages available in the OASIS data. This suggests that the optimal transformation parameters may be quite similar across different scanners and somewhat different T1W imaging sequences.

Rigid body spherical transformation was applied during the alignment of LH and RH in this study. The advantages and disadvantages of linear or non-linear warping procedures have been previously reviewed by Lyttelton et al. [30]. Linear warping often has less statistical power than non-linear warping due to increased local spatial misalignment. However, it has the advantage of computational simplicity and it provides a single unique solution, whereas non-linear procedures are dependent both the template used and on the modeling parameters. In addition, non-linear alignment preserves the original anatomical relationships and coordinate systems in each hemisphere, thus facilitating the comparison with previous results.

## Conclusions

Objective, inter-hemispheric comparisons of anatomical curvature and cortical thickness can be made using a hemispherically-unified coordinate system and visualized on a compact Mollweide map of the entire cortical surface. The unified cortical space preserves virtually all major anatomical features of the two hemispheres, produces accurate co-registration of left- and right hemisphere parcellations over most of the cortex except in areas of high intrinsic anatomical variability and in peri-Sylvian language regions where significant hemispheric asymmetries exist. Analyzing gyral and sulcal structure in this hemispherically unified coordinate system reveals significant differences between the hemispheres in gyral and sulcal structure and in cortical thickness.
